# High-resolution rovibrational and rotational spectroscopy of the singly deuterated cyclopropenyl cation, c-C_3_H_2_D^+^†

**DOI:** 10.1039/d3fd00068k

**Published:** 2023-04-04

**Authors:** Divita Gupta, Weslley G. D. P. Silva, José L. Doménech, Eline Plaar, Sven Thorwirth, Stephan Schlemmer, Oskar Asvany

**Affiliations:** a I. Physikalisches Institut, Universität zu Köln Zülpicher Str. 77 50937 Köln Germany asvany@ph1.uni-koeln.de; b Instituto de Estructura de la Materia, IEM-CSIC Serrano 123 28006 Madrid Spain

## Abstract

Applying a novel action spectroscopic technique in a 4 K cryogenic ion-trap instrument, the molecule c-C_3_H_2_D^+^ has been investigated by high-resolution rovibrational and pure rotational spectroscopy for the first time. In total, 126 rovibrational transitions within the fundamental band of the *ν*_1_ symmetric C–H stretch were measured with a band origin centred at 3168.565 cm^−1^, which were used to predict pure rotational transition frequencies in the ground vibrational state. Based on these predictions, 16 rotational transitions were observed between 90 and 230 GHz by using a double-resonance scheme. These new measurements will enable the first radio-astronomical search for c-C_3_H_2_D^+^.

## Abstract 2

The first rovibrational (IR) and rotational (mm-wave) spectra are measured for c-C_3_H_2_D^+^, a potential detection target in interstellar space.

## Graphical abstract

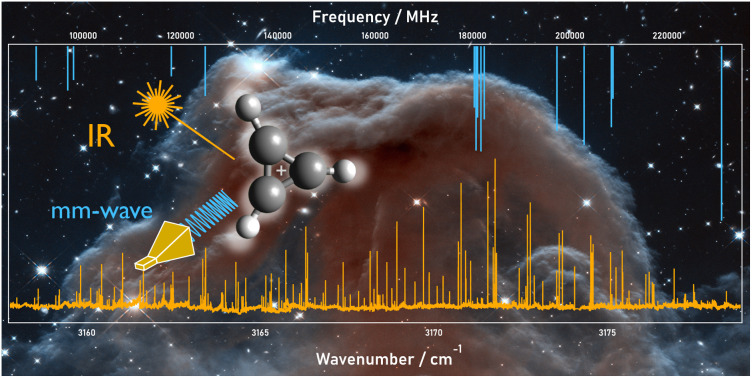

## 1 Introduction

In the late 1970s, a spectral line was detected at 85.3 GHz in a number of different interstellar environments.^1^ Following its firm laboratory identification as the 2_12_ → 1_01_ rotational transition of cyclopropenylidene (c-C_3_H_2_) by Thaddeus *et al.*,^2^ it became clear that this molecule is ubiquitous in space. c-C_3_H_2_ is expected to be formed in the gas phase by the dissociative recombination of the cyclopropenyl cation with an electron: c-C_3_H_3_^+^ + e^−^ → c-C_3_H_2_ + H (see *e.g.* Sipilä *et al.*^3^ and references therein). Cyclopropenylidene is so abundant that even isotopically substituted versions were subsequently detected with a high signal-to-noise ratio, such as the two singly ^13^C-substituted versions,^4^ c-C_3_HD,^5^ and even c-C_3_D_2_.^6^ The deuterated variants of c-C_3_H_2_ are thought to be formed exclusively *via* gas-phase reactions in cold environments, such as the cold cores TMC-1C and L1544, *via* deuterated versions of c-C_3_H_3_^+^, followed again by dissociative recombination. Observational proof of these hypotheses could not be obtained to date, as c-C_3_H_3_^+^ has no permanent electric dipole moment and can thus only be detected in the infrared region.^7^ Its isotopically substituted versions, in particular c-C_3_H_2_D^+^, have a small dipole moment, but high-resolution microwave laboratory data for these are still missing.

In this work, we provide the first rovibrational and pure rotational data for c-C_3_H_2_D^+^, detected by a novel action spectroscopic method. The search for the spectral fingerprints was facilitated by the accurate *ab initio* predictions by Huang and Lee^8^ reported previously.

## 2 Experimental methods

The rovibrational and rotational transitions of c-C_3_H_2_D^+^ were measured in the 4 K 22-pole trap instrument COLTRAP, which has been previously described in detail by Asvany *et al.*^9,10^ and will only be briefly explained here. Ions were generated in a storage ion source by electron impact ionization (*E*_e_ ≈ 30 eV) of a precursor 1 : 1 mixture of singly deuterated acetylene, HCCD (CDN isotopes Inc.; which over time had become a mixture of HCCH, HCCD and DCCD), and methane, CH_4_. This mixture yielded excellent ion production conditions, possibly due to efficient reactions of the form CH_3_^+^ + HCCD → c-C_3_H_2_D^+^ + H_2_ (Ali *et al.*^11^). Every second, a pulse of up to a hundred thousand mass-selected c-C_3_H_2_D^+^ ions (*m* = 40 u) was injected into the 4 K cold 22-pole ion trap. The trap was filled constantly with He (*n* ≈ 10^13^ cm^−3^), and additionally a 1 : 3 Ne : He gas mixture was pulsed into the trap through a piezoelectrically actuated valve at the beginning of each trapping cycle.

Once trapped, the rovibrational transitions of c-C_3_H_2_D^+^ were detected using a novel and very sensitive action spectroscopic method, called leak-out spectroscopy (LOS^12^). This method is based on the escape of a trapped ion after collision-induced transfer of vibrational into kinetic energy. After a cooling period of about 40 ms, the ions were irradiated for 500 ms by an IR beam traversing the trap. During this irradiation time, vibrationally excited c-C_3_H_2_D^+^ ions will eventually be quenched by collisions with the Ne atoms present in the trap. Due to the neutral-to-ion mass ratio of 20 : 40, a substantial part of the vibrational energy is transferred into kinetic energy of the ion, namely a maximum of 20/(20 + 40) × 3170 cm^−1^ = 1057 cm^−1^ ≈ 0.131 eV. By keeping the potential difference between the exit electrode and the floating potential of the trap well below 131 mV, the ions may escape in that direction so that they fly towards the ion detector where they are counted. By repeating these trapping cycles at 1 Hz and counting the escaping c-C_3_H_2_D^+^ ions as a function of the laser wavenumber, a rovibrational spectrum was recorded.

The narrow-band IR radiation was generated by a continuous-wave optical parametric oscillator (cw-OPO, Aculight Argos Model 2400, module C). The IR beam entered the vacuum environment of the ion-trap machine *via* a 0.6 mm thick diamond window (Diamond Materials GmbH), irradiated the trapped ions by crossing the 22-pole trap, and exited the trap instrument *via* a CaF_2_ window, after which it was absorbed by a power meter. The detected power was on the order of 200 mW. The frequency of the IR radiation was measured by a Bristol model 621A wavemeter with an accuracy of about 0.001 cm^−1^ (in well-adjusted settings). We did additional calibration measurements with neutral C_2_H_2_ contained in a gas cell after the IR data reported here was measured. The exact frequencies for C_2_H_2_ are given in the HITRAN database^13^ and, following this calibration, we shifted our data up by 0.007 cm^−1^. With this, the accuracy of the given IR data should be on the order of 0.001 cm^−1^.

For detecting pure rotational transitions, we used a novel double-resonance scheme^14^ based on LOS, as recently described in Asvany *et al.*^15^. A rubidium-clock-referenced microwave synthesizer (Rohde & Schwarz SMF100A) driving an amplifier-multiplier chain (Virginia Diodes Inc. WR9.0M-AMC, WR4.3x2) was used to generate the mm-wave radiation. The radiation was focused by an ellipsoidal mirror (*f* = 43.7 mm (ref. 16)) before entering the vacuum environment through the diamond window. Both the IR and mm-wave radiation sources were used simultaneously, and their beams combined by a small hole in the ellipsoidal mirror through which the narrow IR beam could pass. The frequency of the IR photons is kept fixed on a rovibrational transition starting from a rotational level of the vibrational ground state, resulting in a detectable and constant LOS signal. The mm-wave photon then excites a rotational transition starting or ending on the rotational quantum state probed by the IR laser, thus decreasing or increasing the LOS signal, respectively. A rotational line can therefore be recorded by modulating this LOS signal, *i.e.*, by scanning the frequency of the mm-wave source, as will be shown in section 3.2.

## 3 Experimental measurements

### 3.1 Rovibrational measurements

**Fig. 1:**
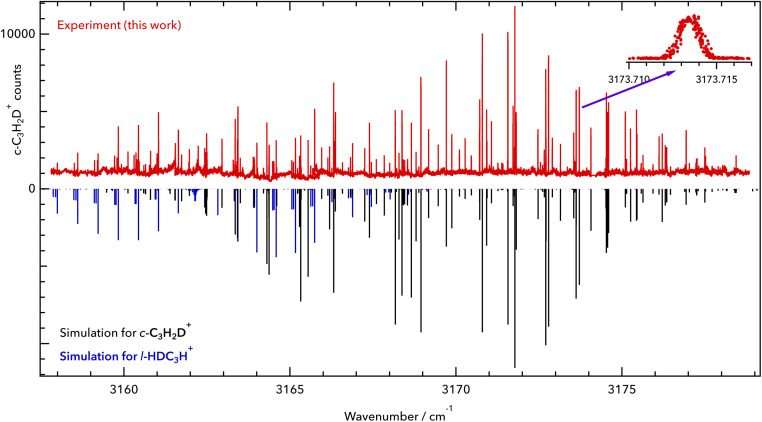
Rovibrational spectrum of the symmetric C–H stretching vibration of c-C_3_H_2_D^+^. Upper part: experimental data of this work (red). Lower part: PGOPHER simulation at a rotational temperature of *T* = 10 K (black sticks). The band origin was found to be at 3168.565 cm^−1^. The zoomed-in inset shows the line 4_13_ ← 3_12_ of the *ν*_1_ = 1 ← 0 band at 3173.714 cm^−1^. Some additional lines have been detected, which are tentatively assigned here to l-HDC_3_H^+^ (blue sticks). The red trace is provided as a separate data file in the ESI.†

c-C_3_H_2_D^+^ is a near-oblate asymmetric top (*C*_2v_ symmetry) with 12 fundamental vibrational modes, none of which have been measured previously in the gas phase, to the best of our knowledge. A previous infrared experimental study for C_3_H_2_D^+^ was done in 1986 by Craig *et al.*^17^ in the solution phase, but its vibrational band centres are subject to shifts. Based on the spectroscopic predictions of Huang and Lee,^8^ we detect the symmetric C–H stretch *ν*_1_ (vibrational symmetry a_1_, a-type transition) at high spectral resolution. The spectrum was measured in the range of our 3 μm OPO over multiple scans ranging over 1.5–2 cm^−1^ and is depicted in Fig. 1. In total, 126 lines were assigned in the *ν*_1_ fundamental band of c-C_3_H_2_D^+^, covering a range of 20 cm^−1^. The observed and assigned line positions are given in the ESI† together with the fit residuals (see section 4 for the final fit).

Apart from the *ν*_1_ lines of c-C_3_H_2_D^+^, additional lines with seemingly characteristic patterns were observed in the spectrum. Based on our mass selectivity (*m* = 40 u) and by considering the possible isomers that could be formed in the source during the ionization of the precursor mixture, we believe that the remaining lines are presumably from singly deuterated l-C_3_H_3_^+^, l-HDC_3_H^+^ or l-H_2_C_3_D^+^. We tentatively assign the additional features to the l-HDC_3_H^+^ form (simulated as blue sticks in Fig. 1), based on theoretical predictions for both isomers of l-C_3_H_2_D^+^ reported by Huang and Lee.^8^ But due to perturbations in the measured spectrum, a definitive assignment needs further investigation, and we postpone the discussion of the l-C_3_H_2_D^+^ species to a forthcoming publication. Additionally, due to the high sensitivity of LOS, some weak lines were seen that could not be assigned to either c-C_3_H_2_D^+^ or l-HDC_3_H^+^. These might be perturbed lines of the l-HDC_3_H^+^ band, or some combination or overtone bands of lower vibrational states of c-C_3_H_2_D^+^.

Because of the low temperature of the ion trap, the transitions exhibit narrow Doppler widths. One example is the line 4_13_ ← 3_12_ of the *ν*_1_ = 1 ← 0 band at 3173.714 cm^−1^, which is depicted in the inset of Fig. 1. This line has a full width at half maximum (FWHM) of about 40 MHz, corresponding to a kinetic temperature of 12 K. This temperature is slightly higher than the nominal trap temperature due to known heating effects.^18^

### 3.2 Rotational measurements

**Fig. 2:**
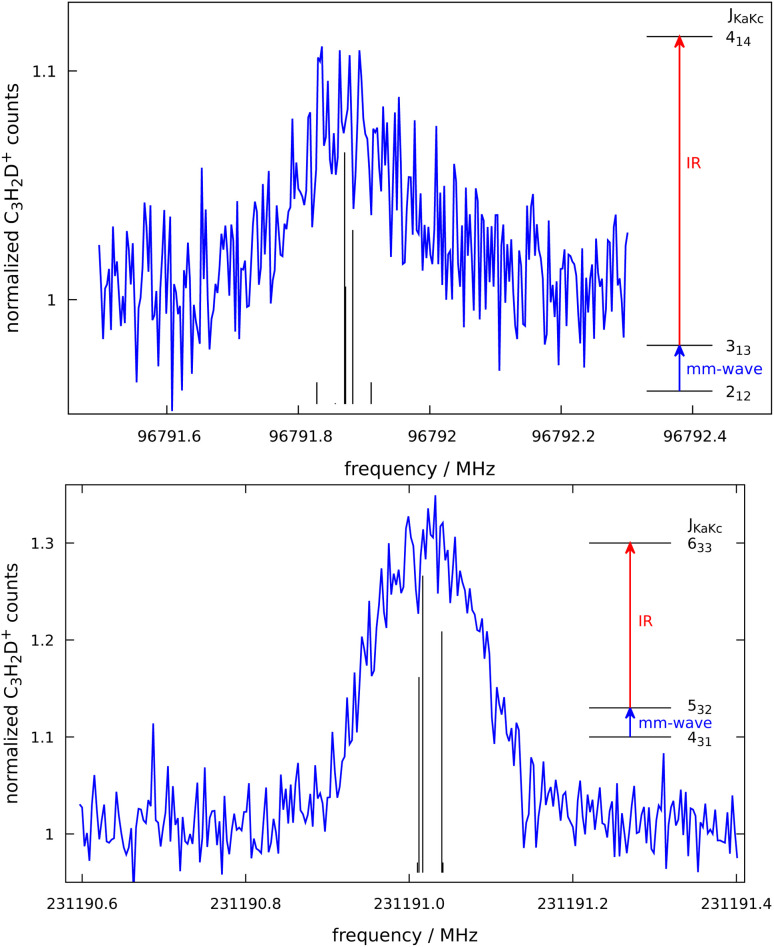
Pure rotational transitions *J*_*K*_a_*K*_c__ = 3_13_ ← 2_12_ and *J*_*K*_a_*K*_c__ = 5_32_ ← 4_31_ of c-C_3_H_2_D^+^ measured by a double-resonance scheme, in which the mm-wave excitation (blue arrows in insets) is followed by IR excitation (red arrows in insets) into the *ν*_1_ vibrational band and subsequent leak-out from the trap. The step sizes are 3 kHz in both cases. The signal counts are normalized and therefore the baseline is unity. The calculated hyperfine structure of the lines are indicated by black sticks. The *J*_*K*_a_*K*_c__ = 3_13_ ← 2_12_ line is power-broadened.

c-C_3_H_2_D^+^ is predicted to have a dipole moment component of 0.225 Debye along its *a*-axis^8^ and should therefore feature weak a-type rotational transitions. Such rotational transitions can be measured in ion traps using double-resonance schemes,^14^ as demonstrated in our group by several examples.^19,20^ The double-resonance scheme using LOS, as applied in this work, has been demonstrated only recently.^15^ Example transitions recorded by applying this double-resonance scheme are shown in Fig. 2.

**Fig. 3:**
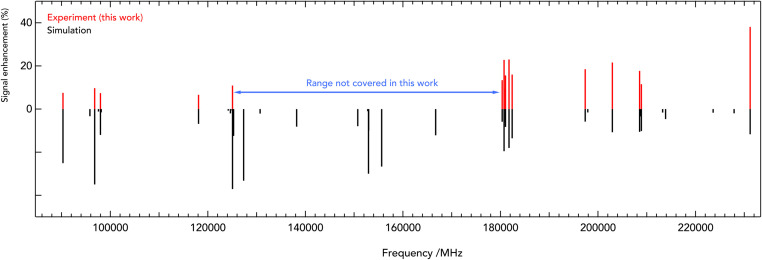
Stick spectra of the pure rotational transitions of c-C_3_H_2_D^+^ measured between 90 and 230 GHz. The experimental frequencies (in red, top) are compared to a PGOPHER simulation at 10 K (black, bottom). The experimental intensities are given as signal enhancement of the normalized ion counts (as in Fig. 2). The regions 90–125 GHz and 175–230 GHz were collected using two different multiplier chain settings and no experimental measurement was done for 125–170 GHz.

Such spectra were recorded in multiple individual measurements in which the mm-wave frequency (blue arrows in Fig. 2) was stepped in an up-and-down manner several times in a given frequency window; the frequency steps were kept constant in individual experiments, and varied between 2 and 10 kHz. Selected rovibrational lines from the *ν*_1_ band were used for the IR excitation (red arrows in Fig. 2). The spectroscopic data were normalized employing a frequency-switching procedure, *i.e.*, by dividing the c-C_3_H_2_D^+^ counts monitored while scanning the spectral window of interest by the counts at an off-resonant mm-wave reference frequency. Therefore, the baselines in Fig. 2 are close to unity. Subsequently, the normalized counts of one measurement were averaged for each given frequency position. The obtained on-resonance signal enhancement is on the order of 8% to 30%, as seen in Fig. 2. Transition frequencies were determined by adjusting the parameters of an appropriate line-shape function (a Gaussian) to the experimental spectrum in a least-squares procedure. In total, 16 rotational lines have been detected, which are summarised in Table 1 and depicted in Fig. 3. The frequencies and their uncertainties in Table 1 result from several (about 3 to 10) independent measurements for each line.

**Table 1:** Measured low-lying rotational transitions of c-C_3_H_2_D^+^ (in MHz). In total, 16 rotational lines have been measured directly by the double resonance technique. Uncertainties are given in parentheses. The obs-calc values are the difference between the observed transition frequency and the calculated frequency using the fitted rotational constants

*J*_*K*_a_ *K*_c__ ← *J*_*K*_a_*K*_c__	This work	obs-calc
2_11_ ← 1_10_	90 293.296(15)	0.0283
3_13_ ← 2_12_	96 791.884(15)	0.0235
3_03_ ← 2_02_	97 972.838(15)	0.0190
3_22_ ← 2_21_	118 087.007(10)	0.0090
4_14_ ← 3_13_	125 054.348(10)	0.0071
5_24_ ← 4_23_	180 356.9896(34)	−0.0024
6_16_ ← 5_15_	180 733.6130(07)	−0.0005
6_06_ ← 5_05_	180 737.9305(16)	0.0021
4_22_ ← 3_21_	181 026.5963(24)	−0.0007
5_14_ ← 4_13_	181 735.1075(07)	−0.0001
4_31_ ← 3_30_	182 399.3395(19)	−0.0002
3_30_ ← 2_11_	197 358.1392(25)	0.0001
5_33_ ← 4_32_	202 928.5429(21)	0.0001
7_17_ ← 6_16_	208 526.1574(26)	0.0003
6_15_ ← 5_14_	208 882.1638(36)	0.0033
5_32_ ← 4_31_	231 191.0231(09)	0.0001

## 4 Spectroscopic parameters

To obtain accurate spectroscopic parameters for the ground and first excited vibrational states of c-C_3_H_2_D^+^, we carried out fits of the experimental transition frequencies using a Watson’s S-reduced Hamiltonian in the *I*^*r*^ representation as implemented in the PGOPHER program.^21^ Initially, the rovibrational lines were assigned using the assistance of *ab initio* calculations from Huang and Lee.^8^ A first fit of these data was then used to predict the pure rotational transition frequencies of c-C_3_H_2_D^+^ in the ground vibrational state. The best-fit ground-state molecular parameters were determined using the pure rotational transition frequencies given in Table 1 together with ground-state combination differences from the IR measurements. As the low-frequency measurements (up to 125 GHz) are affected by power broadening, transition frequencies are thought to be accurate only within 10–15 kHz, while the uncertainties for the measurements at higher frequencies are smaller. The overall rms of the fit is 0.0008 cm^−1^ for the IR combination differences and 11 kHz for the pure rotational data. The complete parameter set is given in Table 2. Hyperfine structure from the presence of the deuterium nucleus (*I*_D_ = 1) was not resolved in our measurements (*cf.*Fig. 2). For the sake of completeness, deuterium (*I*_D_ = 1) quadrupole coupling constants that were estimated at the CCSD(T)/cc-pwCVQZ level of theory (considering all electrons in the correlation treatment) using the CFOUR program^22^ are also given in Table 2. The corresponding structural parameters at this level are *r*_C–C_ = 1.36024 Å and *r*_C–H_ = 1.07828 Å.

**Table 2:** The best-fit spectroscopic parameters (in Watson’s S-reduction) for the ground and first vibrationally excited state (C–H symmetric stretch) of c-C_3_H_2_D^+^ obtained by fitting our data with the program PGOPHER.^21^ All values are in MHz, except for the band origin of *ν*_1_, which is given in cm^−1^. The numbers in parentheses give the uncertainty of the last digits. Our values are compared to the accurate *ab initio* predictions of Huang and Lee^8^

Parameter	Experimental		Calculated^8^	
	Ground state	*ν*_1_	Ground state	*ν*_1_
*ν*_0_	0	3168.56489(19)	0	3164.8/3173.1
*A*	30 747.25030(85)	30 586.58(69)	30 757	30 625
*B*	25 465.51589(40)	25 421.40(56)	25 478	25 442
*C*	13 897.86084(23)	13 859.82(24)	13 901	13 864
*α* ^*A*^		160.67(69)		131.92
*α* ^*B*^		44.12(56)		36.02
*α* ^*C*^		38.04(24)		37.57
*D*_*J*_	0.014734(6)	a	0.0145	
*D*_*JK*_	0.08525(5)	a	0.084	
*D*_*K*_	−0.02599(7)	a	−0.026	
*d*_1_	−0.010248(3)	a	−0.010018	
*d*_2_	−0.005259(3)	a	−0.005106	
*χ*_*aa*_ (D)	0.187^b^			
*χ*_*bb*_ (D)	−0.100^b^			
*χ*_*cc*_ (D)	−0.087^b^			

a The distortion constants of the *ν*_1_ state have been fixed to those determined for the ground state.

b Nuclear quadrupole coupling constants of the deuterium nucleus calculated at the ae-CCSD(T)/cc-pwCVQZ level.

After obtaining the ground-state spectroscopic parameters, the band origin and rotational constants for the *ν*_1_ vibrationally excited state were determined in a second fitting procedure. In this fit, the distortion constants in *ν*_1_ have simply been fixed to those determined for the ground state. The derived spectroscopic parameters for the ground and *ν*_1_ states are summarized in Table 2 along with the theoretical predictions from Huang and Lee.^8^ The line lists of transition frequencies and residuals for both fits are provided in the ESI file.† Overall, the experimentally derived constants show excellent agreement with the calculated values, providing extra confirmation for the unequivocal detection of c-C_3_H_2_D^+^ here.

## 5 Discussion and outlook

Leak-out spectroscopy (LOS) is a novel method for spectroscopy in ion traps, which features general applicability and high sensitivity. This is demonstrated here for the astronomically important c-C_3_H_2_D^+^ molecule, which has evaded spectroscopic detection by other methods so far. The excellent signal quality (the background in Fig. 1 is on the order of 1000 c-C_3_H_2_D^+^ counts per trapping cycle, while the signal is on the order of 11 000 counts for the strongest rovibrational lines) allowed the complete IR spectrum, shown in Fig. 1, to be recorded within a few days. Also, subsequent measurements of its 16 rotational lines in the ground state provide the first experimental rotational spectrum, which will be useful for the astronomical community.

As mentioned in the introduction, c-C_3_H_2_ is a molecule relevant for the chemistry of cold interstellar environments,^23,24^ which is thought to be formed from c-C_3_H_3_^+^ by dissociative recombination with an electron. But while the abundant c-C_3_H_2_ has a very large permanent dipole moment of 3.43 D (ref. 25) and was thus detected in the early days of radio astronomy, c-C_3_H_3_^+^ (*D*_3h_ point-group symmetry) has no permanent dipole moment making its identification through rotational spectroscopy or radio astronomy impossible. The recently launched James Webb Space Telescope (JWST) offers higher sensitivity for the infrared region, which might aid the detection of c-C_3_H_3_^+^ using its high-resolution rovibrational spectrum.^7^

Inclusion of one ^13^C or one D atom in the structure of c-C_3_H_3_^+^ imparts a non-zero dipole moment component in the isotopologues. In the case of c-C_3_H_2_D^+^, this dipole moment is calculated to be 0.225 D,^8^ which opens up the possibility for its search in various interstellar environments by its rotational fingerprints. This may be especially relevant for regions where other carbocations, such as l-C_3_H^+^ and the neutral c-C_3_H_2_ (and its isotopologues), have been detected.^6,26,27^ These future detections could provide crucial inputs to improve low-temperature chemical models, and potentially highlight which reactions and their deuterated versions play a significant role in the hydrocarbon chemistry in these environments.

Among the different c-C_3_H_3_^+^ isotopologues, given the dipole moment and elemental isotope abundances, it would seem that c-C_3_H_2_D^+^ should be the easiest species to be identified in an astronomical spectrum. It is worth noting that its dipole moment is essentially twice that of carbon monoxide, which has a dipole moment of only 0.122 D.^24^ For the warm carbon chain chemistry in the protostar L1527, a column density of ∼10^12^ cm^−2^ is determined for c-C_3_HD by Sakai *et al.*,^26^ implying a column density of ∼10^10^ cm^−2^ for c-C_3_H_2_D^+^ (Aikawa, private communication, based on her model^28^). Similarly, for the colder pre-stellar core L1544, based on the abundance of c-C_3_HD (∼4–6 × 10^12^ cm^−2^; Spezzano *et al.*,^6^ and Giers *et al.*^29^), and available astrochemical models,^3,28^ a column density in the order of 3 × 10^10^ cm^−2^ is estimated for c-C_3_H_2_D^+^. This low column density in combination with the small dipole moment and the diluted spectral signature of this asymmetric top molecule results in integration times for strong selected lines in excess of several hundred hours for a single-dish telescope such as the IRAM 30 m facility (Schilke, Kim; private communication), rendering its current radio-astronomical detection very challenging. Indeed, this molecule has not been found in the deep surveys QUIJOTE^31^ and GOTHAM^32^ of TMC-1 (Cernicharo and McGuire, respectively, private communication).

Nevertheless, the first rotational spectra for this molecule obtained here lays the groundwork for future astronomical searches. The high sensitivity of the novel LOS method, with the possibility to be used also in an infrared-millimetre-wave double-resonance fashion, will also enable new investigations of challenging molecular ions relevant to astrochemistry. A recent example from our laboratory concerns another member of the C_3_H_3_^+^ family, namely open-chain H_2_C_3_H^+^, which is thought to be the precursor for l-C_3_H_2_ in space.^30^ A publication about its rovibrational and rotational signatures is in preparation.

## Author contributions

Divita Gupta: writing – original draft, methodology, investigation, formal analysis, visualization, validation, writing – review & editing. Weslley G. D. P. Silva: writing – original draft, formal analysis, investigation, visualization, validation, writing – review & editing. José L. Doménech: investigation, validation, writing – review & editing. Eline Plaar: investigation, writing – review & editing. Sven Thorwirth: validation, writing – review & editing. Stephan Schlemmer: conceptualization, funding acquisition, project administration, supervision, writing – review & editing. Oskar Asvany: conceptualization, funding acquisition, validation, methodology, supervision, writing – original draft, writing – review & editing.

## Conflicts of interest

There are no conflicts to declare.

## Supplementary Material

FD-245-D3FD00068K-s001
