# Supplementary material for: High-resolution rovibrational and rotational spectroscopy of the singly deuterated cyclopropenyl cation, c-C3H2D+
Source: Faraday Discuss. 2023 Apr 4;245:298–308. doi: 10.1039/d3fd00068k (PMC10510037; doi:10.1039/d3fd00068k)
Supplement: FD-245-D3FD00068K-s001 [file FD-245-D3FD00068K-s001.zip › suppl_mat.pdf]

Supplementary Material: High-resolution rovibrational and rotational spectroscopy of singly deuterated cyclopropenyl cation, c-$C_3H_2D^+$

Divita Gupta,† Weslley G. D. P. Silva,† José L. Doménech,‡ Eline Plaar,† Sven Thorwirth,† Stephan Schlemmer,† and Oskar Asvany*,†

†*I. Physikalisches Institut, Universität zu Köln Zülpicher Str. 77, 50937 Köln, Germany*

‡*Instituto de Estructura de la Materia, IEM-CSIC, Serrano 123, 28006 Madrid, Spain*

E-mail: asvany@ph1.uni-koeln.de

Transition Frequencies and Residuals (in wavenumbers) for the First Vibrationally Excited State Fit

Observed	Obs-Calc	v’	J’	Ka’	Kc’		v"	J"	Ka"	Kc"
3160.1209	0.0004	1	3	1	2	-	0	4	3	1
3160.6526	0.0001	1	5	3	3	-	0	6	3	4
3160.8028	0.0031	1	4	3	1	-	0	5	3	2
3160.9529	0.0020	1	2	1	2	-	0	3	3	1
3161.5372	0.0017	1	5	1	4	-	0	6	1	5
3161.5470	0.0018	1	6	0	6	-	0	7	0	7
3161.5470	0.0018	1	6	1	6	-	0	7	1	7
3161.7347	-0.0001	1	4	3	2	-	0	5	3	3
3161.9547	0.0013	1	2	0	2	-	0	3	2	1
3161.9702	0.0010	1	2	1	1	-	0	3	3	0
3162.4331	0.0013	1	3	3	0	-	0	4	3	1
3162.4617	0.0011	1	4	1	3	-	0	5	1	4
3162.4908	0.0015	1	5	1	5	-	0	6	1	6
3162.4908	0.0016	1	5	0	5	-	0	6	0	6
3162.4990	0.0012	1	3	2	1	-	0	4	2	2
3162.5041	0.0007	1	4	2	3	-	0	5	2	4
3162.9534	0.0007	1	3	3	1	-	0	4	3	2
3163.3498	0.0005	1	3	1	2	-	0	4	1	3
3163.4326	0.0014	1	4	1	4	-	0	5	1	5
3163.5022	-0.0023	1	3	2	2	-	0	4	2	3
3163.9320	-0.0006	1	2	2	0	-	0	3	2	1
3164.2014	-0.0007	1	1	0	1	-	0	2	2	0
3164.3047	-0.0002	1	2	1	1	-	0	3	1	2

3164.3616	0.0007	1	5	1	5	-	0	5	1	4
3164.3662	0.0001	1	3	0	3	-	0	4	0	4
3164.3729	0.0001	1	3	1	3	-	0	4	1	4
3165.2300	0.0005	1	5	1	4	-	0	5	3	3
3165.2789	0.0007	1	4	0	4	-	0	4	2	3
3165.2865	-0.0004	1	2	0	2	-	0	3	0	3
3165.3246	0.0002	1	2	1	2	-	0	3	1	3
3165.3368	0.0006	1	4	1	4	-	0	4	1	3
3165.5457	-0.0005	1	1	1	0	-	0	2	1	1
3165.9567	0.0007	1	4	1	3	-	0	4	3	2
3166.1406	0.0008	1	3	0	3	-	0	3	2	2
3166.1937	-0.0000	1	1	0	1	-	0	2	0	2
3166.3181	0.0000	1	1	1	1	-	0	2	1	2
3166.3438	0.0004	1	3	1	2	-	0	3	3	1
3166.3687	0.0009	1	3	1	3	-	0	3	1	2
3166.5814	0.0009	1	4	2	3	-	0	4	2	2
3166.8213	0.0005	1	2	0	2	-	0	2	2	1
3166.8855	0.0022	1	5	3	3	-	0	5	3	2
3167.2517	-0.0001	1	0	0	0	-	0	1	0	1
3167.3958	0.0004	1	2	1	2	-	0	2	1	1
3167.6008	-0.0032	1	3	2	2	-	0	3	2	1
3167.8399	-0.0016	1	4	3	2	-	0	4	3	1
3168.1715	-0.0009	1	1	1	1	-	0	1	1	0
3168.2828	-0.0004	1	2	2	1	-	0	2	2	0
3168.3748	0.0001	1	3	3	1	-	0	3	3	0
3168.4029	0.0012	1	5	5	1	-	0	5	5	0
3168.4136	0.0008	1	4	4	1	-	0	4	4	0

3168.4549	0.0013	1	5	5	0	-	0	5	5	1
3168.5410	0.0006	1	4	4	0	-	0	4	4	1
3168.6553	0.0007	1	3	3	0	-	0	3	3	1
3168.7990	-0.0011	1	2	2	0	-	0	2	2	1
3168.8807	-0.0022	1	5	4	1	-	0	5	4	2
3168.9434	-0.0005	1	1	1	0	-	0	1	1	1
3169.1752	0.0016	1	4	3	1	-	0	4	3	2
3169.4651	0.0003	1	3	2	1	-	0	3	2	2
3169.7096	-0.0003	1	2	1	1	-	0	2	1	2
3169.8744	-0.0008	1	1	0	1	-	0	0	0	0
3170.1039	0.0001	1	5	3	2	-	0	5	3	3
3170.2742	-0.0005	1	2	2	1	-	0	2	0	2
3170.4606	-0.0010	1	4	2	2	-	0	4	2	3
3170.7100	-0.0004	1	3	3	1	-	0	3	1	2
3170.7161	-0.0007	1	3	1	2	-	0	3	1	3
3170.7932	0.0000	1	2	1	2	-	0	1	1	1
3170.9226	-0.0007	1	2	0	2	-	0	1	0	1
3170.9340	-0.0034	1	3	2	2	-	0	3	0	3
3171.0687	-0.0016	1	4	3	2	-	0	4	1	3
3171.2624	-0.0010	1	6	3	3	-	0	6	3	4
3171.3778	-0.0002	1	4	1	3	-	0	3	3	0
3171.3817	-0.0015	1	5	4	2	-	0	5	2	3
3171.4133	-0.0000	1	4	4	1	-	0	4	2	2
3171.5546	-0.0003	1	5	2	3	-	0	5	2	4
3171.5635	-0.0007	1	2	1	1	-	0	1	1	0
3171.7187	-0.0000	1	4	1	3	-	0	4	1	4
3171.7609	-0.0008	1	5	3	3	-	0	5	1	4

3171.7728	-0.0001	1	3	1	3	-	0	2	1	2
3171.8124	-0.0003	1	3	0	3	-	0	2	0	2
3172.4680	-0.0034	1	3	2	2	-	0	2	2	1
3172.6078	-0.0007	1	6	3	4	-	0	6	1	5
3172.6600	0.0000	1	5	1	4	-	0	5	1	5
3172.6703	-0.0000	1	5	2	4	-	0	5	0	5
3172.7040	0.0002	1	4	1	4	-	0	3	1	3
3172.7113	0.0002	1	4	0	4	-	0	3	0	3
3172.7871	-0.0008	1	3	1	2	-	0	2	1	1
3172.9009	-0.0016	1	2	2	0	-	0	1	0	1
3173.1462	0.0000	1	3	2	1	-	0	2	2	0
3173.5469	-0.0006	1	4	2	3	-	0	3	2	2
3173.5778	-0.0000	1	6	1	5	-	0	6	1	6
3173.6197	0.0005	1	5	1	5	-	0	4	1	4
3173.6197	-0.0006	1	5	0	5	-	0	4	0	4
3173.7135	-0.0003	1	4	1	3	-	0	3	1	2
3174.0632	-0.0012	1	4	3	2	-	0	3	3	1
3174.4875	-0.0007	1	7	1	6	-	0	7	1	7
3174.5186	0.0002	1	5	2	4	-	0	4	2	3
3174.5297	0.0000	1	6	0	6	-	0	5	0	5
3174.5297	0.0002	1	6	1	6	-	0	5	1	5
3174.5604	-0.0006	1	4	2	2	-	0	3	2	1
3174.5653	0.0002	1	5	1	4	-	0	4	1	3
3174.5967	0.0010	1	4	3	1	-	0	3	3	0
3175.0995	0.0004	1	3	3	0	-	0	2	1	1
3175.1377	0.0000	1	3	2	1	-	0	2	0	2
3175.2562	-0.0010	1	5	3	3	-	0	4	3	2

3175.4361	-0.0009	1	7	1	7	-	0	6	1	6
3175.4361	-0.0009	1	7	0	7	-	0	6	0	6
3175.4403	0.0001	1	6	2	5	-	0	5	2	4
3175.4496	0.0001	1	6	1	5	-	0	5	1	4
3175.5775	-0.0012	1	5	4	2	-	0	4	4	1
3175.6321	0.0001	1	5	2	3	-	0	4	2	2
3175.9322	-0.0011	1	5	4	1	-	0	4	4	0
3176.1160	0.0006	1	3	3	1	-	0	2	1	2
3176.2112	0.0006	1	5	3	2	-	0	4	3	1
3176.3026	0.0001	1	6	3	4	-	0	5	3	3
3176.3401	-0.0018	1	8	1	8	-	0	7	1	7
3176.3401	-0.0018	1	8	0	8	-	0	7	0	7
3176.3479	0.0015	1	7	2	6	-	0	6	2	5
3176.3479	-0.0000	1	7	1	6	-	0	6	1	5
3176.4616	0.0004	1	6	2	4	-	0	5	2	3
3176.9325	0.0012	1	4	3	1	-	0	3	1	2
3177.0276	0.0005	1	6	5	2	-	0	5	5	1
3177.2234	-0.0023	1	6	5	1	-	0	5	5	0
3177.4940	-0.0003	1	6	3	3	-	0	5	3	2
3177.5173	0.0007	1	4	4	0	-	0	3	2	1
3177.7160	0.0018	1	6	4	2	-	0	5	4	1
3177.8950	0.0007	1	4	2	2	-	0	3	0	3

Ground State Rotational Transition Frequencies and Residuals (in MHz)

Observed	Obs-Calc	v'	J'	Ka'	Kc'		v"	J"	Ka"	Kc"
90293.2964	0.0283	0	2	1	1	-	0	1	1	0
96791.8844	0.0235	0	3	1	3	-	0	2	1	2
97972.8381	0.0190	0	3	0	3	-	0	2	0	2
118087.0068	0.0090	0	3	2	2	-	0	2	2	1
125054.3480	0.0071	0	4	1	4	-	0	3	1	3
180356.9896	-0.0024	0	5	2	4	-	0	4	2	3
180733.6130	-0.0005	0	6	1	6	-	0	5	1	5
180737.9305	0.0021	0	6	0	6	-	0	5	0	5
181026.5963	-0.0007	0	4	2	2	-	0	3	2	1
181735.1075	-0.0001	0	5	1	4	-	0	4	1	3
182399.3395	-0.0002	0	4	3	1	-	0	3	3	0
197358.1392	0.0001	0	3	3	0	-	0	2	1	1
202928.5429	0.0001	0	5	3	3	-	0	4	3	2
208526.1590	0.0003	0	7	1	7	-	0	6	1	6
208882.1686	0.0033	0	6	1	5	-	0	5	1	4
231191.0231	0.0001	0	5	3	2	-	0	4	3	1

Ground state combination differences and residuals (in wavenumbers)

Observed	Obs-Calc	v'	J'	Ka'	Kc'		v"	J"	Ka"	Kc'
3.6807	-0.0008	0	2	0	2	-	0	0	0	0
1.9922	0.0007	0	2	2	0	-	0	2	0	2
1.8534	-0.0009	0	2	1	2	-	0	1	1	0
3.3977	-0.0001	0	2	1	1	-	0	1	1	1
4.1013	-0.0011	0	2	2	1	-	0	1	0	1
1.5349	0.0008	0	3	0	3	-	0	2	2	1
3.3318	-0.0016	0	3	2	1	-	0	3	0	3
4.1019	-0.0005	0	2	2	1	-	0	1	0	1
4.8670	-0.0005	0	3	2	1	-	0	2	2	1
1.9915	-0.0001	0	2	2	0	-	0	2	0	2
1.8539	-0.0004	0	2	1	2	-	0	1	1	0
5.4049	-0.0001	0	3	1	2	-	0	2	1	2
2.3345	-0.0012	0	3	3	0	-	0	3	1	2
3.3974	-0.0003	0	2	1	1	-	0	1	1	1
2.0712	0.0001	0	3	1	3	-	0	2	1	1
4.3717	-0.0017	0	3	3	1	-	0	3	1	3
1.5341	0.0000	0	3	0	3	-	0	2	2	1
3.3332	-0.0003	0	3	2	1	-	0	3	0	3
4.0986	-0.0008	0	4	2	3	-	0	3	2	1
5.6719	-0.0010	0	3	2	2	-	0	2	0	2
1.7744	0.0007	0	4	0	4	-	0	3	2	2
1.9915	-0.0000	0	2	2	0	-	0	2	0	2
3.6811	-0.0003	0	3	2	2	-	0	2	2	0
6.9661	-0.0008	0	4	2	2	-	0	3	2	2

5.4040	-0.0010	0	3	1	2	-	0	2	1	2
1.9958	0.0008	0	4	1	4	-	0	3	1	2
5.4060	0.0010	0	3	1	2	-	0	2	1	2
2.3352	-0.0005	0	3	3	0	-	0	3	1	2
5.4214	-0.0006	0	4	3	2	-	0	3	3	0
2.0710	-0.0001	0	3	1	3	-	0	2	1	1
4.3723	-0.0011	0	3	3	1	-	0	3	1	3
2.9940	-0.0001	0	4	1	3	-	0	3	3	1
3.2288	0.0001	0	4	3	1	-	0	4	1	3
6.4442	-0.0003	0	3	3	1	-	0	2	1	1
6.2222	-0.0006	0	4	3	1	-	0	3	3	1
7.4324	-0.0005	0	4	2	3	-	0	3	0	3
3.3347	0.0013	0	3	2	1	-	0	3	0	3
4.0998	0.0004	0	4	2	3	-	0	3	2	1
8.9763	0.0002	0	4	4	1	-	0	3	2	1
6.9655	-0.0014	0	4	2	2	-	0	3	2	2
4.0773	0.0002	0	5	2	4	-	0	4	2	2
2.9997	-0.0008	0	4	4	0	-	0	4	2	2
2.3356	-0.0001	0	3	3	0	-	0	3	1	2
0.3409	0.0002	0	3	3	0	-	0	4	1	4
5.7620	-0.0008	0	4	3	2	-	0	4	1	4
3.4950	-0.0004	0	5	1	4	-	0	4	3	2
2.3359	0.0002	0	3	3	0	-	0	3	1	2
5.4215	-0.0006	0	4	3	2	-	0	3	3	0
8.3724	-0.0015	0	5	3	2	-	0	4	3	2
7.3671	-0.0004	0	4	1	3	-	0	3	1	3
1.9043	-0.0008	0	5	1	5	-	0	4	1	3

2.9945	0.0004	0	4	1	3	-	0	3	3	1
3.2287	-0.0000	0	4	3	1	-	0	4	1	3
6.1053	-0.0015	0	5	3	3	-	0	4	3	1
1.8483	0.0002	0	5	0	5	-	0	4	2	3
4.1958	0.0003	0	5	2	3	-	0	4	4	1
11.1288	-0.0022	0	6	0	6	-	0	4	0	4
4.0775	0.0004	0	5	2	4	-	0	4	2	2
7.0515	0.0011	0	5	4	2	-	0	4	4	0
9.2580	-0.0001	0	5	1	4	-	0	4	1	4
1.8708	-0.0009	0	6	1	6	-	0	5	1	4
3.4953	-0.0002	0	5	1	4	-	0	4	3	2
4.8754	-0.0031	0	5	3	2	-	0	5	1	4
6.2330	0.0021	0	6	3	4	-	0	5	3	2
1.9053	0.0002	0	5	1	5	-	0	4	1	3
7.4300	-0.0005	0	5	3	3	-	0	5	1	5
3.6928	-0.0012	0	6	1	5	-	0	5	3	3
6.1073	0.0005	0	5	3	3	-	0	4	3	1
12.9827	-0.0018	0	7	0	7	-	0	5	0	5
1.8718	0.0001	0	6	1	6	-	0	5	1	4
6.2316	0.0007	0	6	3	4	-	0	5	3	2
12.9827	-0.0016	0	7	1	7	-	0	5	1	5
3.6948	0.0008	0	6	1	5	-	0	5	3	3
1.8605	0.0007	0	7	1	7	-	0	6	1	5
